# Deep learning of bone metastasis in small cell lung cancer: A large sample-based study

**DOI:** 10.3389/fonc.2023.1097897

**Published:** 2023-01-27

**Authors:** Qing Chen, Haifeng Liang, Lei Zhou, Hongwei Lu, Fancheng Chen, Yuxiang Ge, Zhichao Hu, Ben Wang, Annan Hu, Wei Hong, Libo Jiang, Jian Dong

**Affiliations:** ^1^ Department of Orthopedic Surgery, Zhongshan Hospital, Fudan University, Shanghai, China; ^2^ Cancer Center, Zhongshan Hospital, Fudan University, Shanghai, China; ^3^ Department of Orthopedics Surgery, Minhang Hospital, Fudan University, Shanghai, China; ^4^ Department of Orthopedic Surgery, Zhongshan Hospital Wusong Branch, Fudan University, Shanghai, China; ^5^ Department of Geriatrics and Gerontology, Huadong Hospital, Fudan University, Shanghai, China; ^6^ Shanghai Key Laboratory of Clinical Geriatric Medicine, Huadong Hospital, Fudan University, Shanghai, China

**Keywords:** small cell lung cancer, bone metastasis, survival, nomogram, decision tree

## Abstract

**Introduction:**

Bone is a common metastatic site for small cell lung cancer (SCLC). Bone metastasis (BM) in patients have are known to show poor prognostic outcomes. We explored the epidemiological characteristics of BM in SCLC patients and create a new deep learning model to predict outcomes for cancer-specific survival (CSS) and overall survival (OS).

**Materials and Methods:**

Data for SCLC patients diagnosed with or without BM from 2010 to 2016 were retrieved from the Surveillance, Epidemiology, and End Results (SEER) database. Univariate and multivariate Cox proportional hazards regression models were used to evaluate the effects of prognostic variables on OS and CSS. Through integration of these variables, nomograms were created for the prediction of CSS and OS rates at 3-month,6- month,and 12-month. Harrell's coordination index, calibration curves,and time- dependent ROC curves were used to assess the nomograms' accuracy. Decision tree analysis was used to evaluate the clinical application value of the established nomogram.

**Results:**

In this study, 4201 patients were enrolled. Male sex, tumor size 25 but <10, brain and liver metastases, as well as chemotherapy were associated with a high risk for BM. Tumor size, Age, N stage, gender, liver metastasis, radiotherapy as well as chemotherapy were shown to be prognostic variables for OS, and the prognostic variables for CSS were added to the tumor number in addition. Based on these results, nomograms for CSS and OS were established separately. Internal as well as external validation showed that the C-index, calibration cuurve and DCA had good constructive correction effect and clinical application value. Decision tree analysis further confirmed the prognostic factors of OS and CSS.

**Discussion:**

The nomogram and decision tree models developed in this study effectively guided treatment decisions for SCLC patients with BM. The creation of prediction models for BM SCLC patients may be facilitated by deep learning models.

## 1 Introduction

The majority of deaths from cancer are caused by lung cancer, one of the most common malignancies (1). Annually, lung cancer incidences are approximately 53.6/100,000 with a mortality rate of 45.6/100,000 (2). Small cell lung cancer (SCLC) accounts for about 15-20% of lung cancer cases, characterized by high malignancy, distant metastasis, and poor survival outcomes (3). Based on the Veterans Administration Lung Study Group approach, SCLC is staged using a binary two-stage categorization system—limited disease (LD) and extensive disease (ED). The five-year survival rate for LD patients is 2%, while the 3-year survival rate for ED patients is 1%–15% (4–6).

SCLC as a malignant tumor has the ability of high and multisite metastases, especially to the bone, liver, and brain (7). Compared to prostate and breast carcinomas, SCLC is highly metastatic with poorer survival outcomes. Once bone metastases (BM) occur, patients will have significantly shorter overall survival (OS) time, which has been shown to be cut in half. Additionally, it may cause significant morbidity, such as spinal cord compression, pathological fractures, and bone discomfort that lower quality of life (8).

No major advances in the treatment of SCLC have been established in the past 30 years. Researchers have been forced to acknowledge that progress in SCLC treatment is behind of non-small cell lung cancer (NSCLC) and many other carcinomas, particularly in the development of targeted therapies and molecular profiling (9). For limited-stage SCLC, thoracic radiotherapy combined with platinum-based chemotherapy is currently advised, but for ED, chemotherapy alone is advised with prophylactic cranial irradiation due to early SCLC spread and its chemo sensitive characteristics (10). Moreover, for stage I (limited) SCLC, surgery, followed by adjuvant chemotherapy has been recommended. Immunotherapy, such as one targeting PD-1/PD-L1, has already changed the clinical treatment of SCLC and is recommended by the National Comprehensive Cancer Network (NCCN), based on their guidelines (11, 12).

Although there have been some studies on non-SCLC BM, epidemiology and signatures of SCLC with BM are still unclear (13, 14), and for SCLC patients diagnosed with BM, no clinical prediction model has been developed to predict their survival outcomes. Hence, we aim to answer the following (1): Which clinicopathological features are risk factors for BM in SCLC patients? (2) Which clinicopathological features are independent survival predictors for SCLC patients with BM? (3) Can nomograms and decision trees be created based on recognized prognostic markers for accurate prediction of OS and CSS in patients with SCLC and BM?

To address these unresolved issues, we utilized the large, population-centered SEER database to investigate applications of deep learning in the prevention, diagnosis, as well as BM treatment in SCLC.

## 2 Materials and methods

### 2.1 Data retrieval

Data was obtained from the most current publication of the SEER 18 registry database, which had been made available in November 2018. The SEER 18 database owned by the National Cancer Institute provided the largest source of registry data on tumor incidences as well as survival rates between 2010 and 2016, which would be appropriate for this study. However, data on metastatic bone, brain and liver sites were not documented until 2010, so we included patients diagnosed with SCLC after 2010. Because the data from the SEER database were publicly accessible and de-identified, ethical approval and the need for informed consents were waived.

### 2.2 Cohort selection

The inclusion criteria for this study were (1): Between 2010 and 2016, cytology or biopsy samples were used to establish pathologic diagnosis under a microscope. Patients who had been diagnosed before 2010 were not included (2). SCLC diagnosis was based on the International Classification of Diseases for Oncology, 3rd Edition (ICD-3) histology codes 8041, 8042, 8043, 8044, and 8045 (3). Site as well as morphology were recoded as ICD-O-3/WHO 2008: lung and bronchus

**Figure 1:**
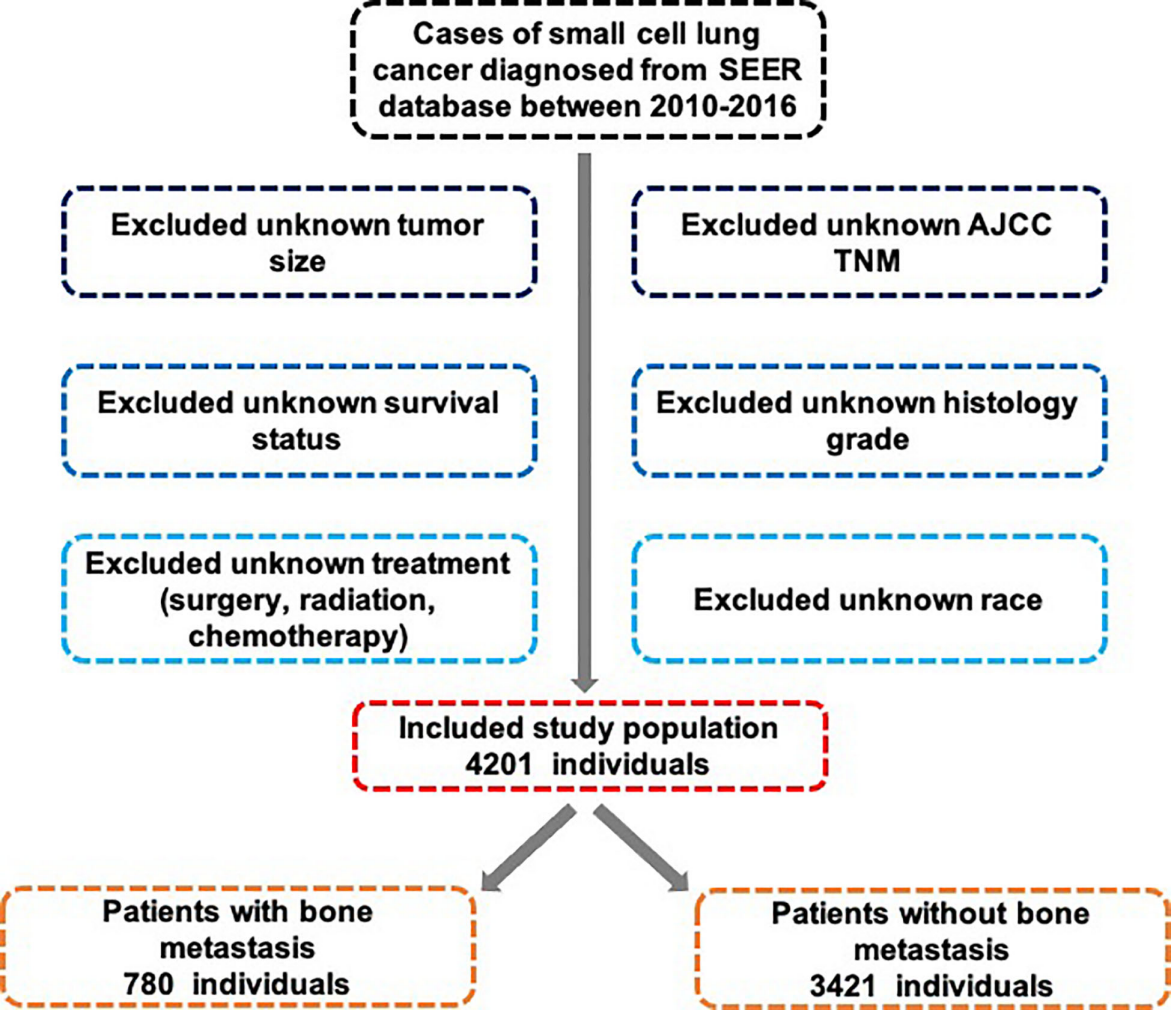
Deep learning process for SCLC patients with BM.

Exclusion criteria were (1): death certificate or autopsy did not confirm SCLC and (2) incomplete follow-up information (**Figure 1**).

### 2.3 Data elements

Relevant variables were extracted from each patient’s data. Age, sex, race, and survival time (months) were included. Pathological variables were the tumor number, primary site, tumor size, T stage, laterality, N stage and grade. In addition, clinical characteristics, including radiation sequence with surgery, surgery, radiation, and chemotherapy, were collected from the SEER database. The specific variable information is shown in **Table 1**.

**Table 1:** Baseline demographics and clinicopathological characteristics of SCLC patients.

Characteristics	Total (n=4201)	Total patients with SCLC	
		With bone metastasis (%)	Without bone metastasis (%)
Age group			
<65	1429(34.02%)	279(6.64%)	1150(27.37%)
≥65	2772(65.92%)	501(11.93%)	2271(54.06%)
Gender			
Male	1998(47.56%)	421(10.02%)	1577(37.54%)
Female	2103(52.44%)	359(8.55%)	1844(43.89%)
Race			
Black	265(6.31%)	42(1.00%)	223(5.31%)
White	3747(89.19%)	707(16.83%)	3040(72.36%)
Other	189(4.50%)	31(0.74%)	158(3.76%)
Histologic type			
Small cell carcinoma, NOS	3863(91.95%)	736(17.52%)	3127(74.43%)
Oat cell carcinoma	112(2.67%)	19(0.45%)	93(2.21%)
Small cell carcinoma, fusiform cell	3(0.07%)	1(0.02%)	2(0.05%)
Small cell carcinoma, intermediate cell	10(0.26%)	3(0.07%)	7(0.17%)
Combined small cell carcinoma	213(5.07%)	21(0.50%)	192(4.57%)
Tumor number			
N=1	3137(74.67%)	628(14.95%)	2509(59.72%)
N>1	1064(25.33%)	152(3.62%)	912(21.71%)
Primary site			
Main bronchus	407(9.69%)	88(2.09%)	319(7.59%)
Upper lobe, lung	390(52.58%)	390(9.28%)	1819(43.30%)
Middle lobe, lung	192(4.57%)	32(0.76%)	160(3.81%)
Lower lobe, lung	1066(25.37%)	201(4.78%)	865(20.59%)
Overlapping leision of lung	72(1.71%)	12(0.29%)	60(1.43%)
Lung, NOS	255(6.07%)	57(1.36%)	198(4.71%)
Laterality			
Bilateral	15(0.36%)	4(0.10%)	11(0.26%)
Left-origin of primary	1837(43.73%)	327(7.78%)	1510(35.94%)
Right-origin of primary	2349(55.92%)	449(10.69%)	1900(45.23%)
Grade			
I	26(0.62%)	5(0.12%)	21(0.50%)
II	33(1.37%)	2(0.05%)	31(0.74%)
III	1545(36.78%)	274(6.52%)	1271(30.25%)
IV	2597(61.82%)	499(11.88%)	2098(49.94%)
Size group			
0≤X<3	1176(27.99%)	152(3.62%)	1024(24.38%)
3≤X<5	896(21.33%)	217(5.17%)	879(20.92%)
5≤X<7	853(20.30%)	208(4.95%)	645(15.35%)
7≤X<10	748(17.81%)	138(3.28%)	610(14.52%)
X≥10	328(7.81%)	65(1.55%)	263(6.26%)
T			
T1	709(16.88%)	71(1.69%)	638(15.19%)
T2	1127(26.83%)	189(4.50%)	938(22.33%)
T3	938(22.33%)	191(4.55%)	747(17.78%)
T4	1325(31.54%)	299(7.12%)	1026(2.44%)
TX	102(2.43%)	30(0.71%)	72(1.71%)
N			
N0	929(22.11%)	68(1.62%)	861(20.50%)
N1	410(9.76%)	56(1.33%)	354(8.43%)
N2	2087(49.68%)	427(10.16%)	1660(39.51%)
N3	697(16.59%)	212(5.05%)	485(11.54%)
NX	77(18.33%)	17(0.40%)	61(1.45%)
Brain metastasis			
No	3589(85.43%)	631(15.02%)	2958(70.41%)
Yes	612(14.57%)	149(3.55%)	463(11.02%)
Liver metastasis			
No	3248(77.31%)	365(8.69%)	2883(68.63%)
Yes	953(22.69%)	415(9.88%)	538(12.81%)
Radiation sequence with surgery			
No	3776(89.88%)	729(17.35%)	3047(72.53%)
After	408(9.71%)	47(1.12%)	361(8.59%)
Prior	17(0.41%)	4(0.10%)	13(0.31%)
Surgery			
No/Unknown	3787(90.15%)	770(18.33%)	3017(71.82%)
Yes	414(9.85%)	10(0.24%)	404(9.63%)
Radiation			
No/Unknown	2148(51.13%)	453(10.78%)	1695(40.35%)
Yes	2053(48.87%)	327(7.78%)	1726(41.09%)
Chemotherapy			
No/Unknown	1200(28.56%)	201(4.78%)	999(23.78%)
Yes	3001(71.44%)	579(13.78%)	2422(57.65%)

In order to get all of the raw data for this study from the SEER website (https://seer.cancer.gov/data), we submitted an access request, agreement, and signed the SEER research.

### 2.4 Study outcome

The OS outcome was determined as the survival interval from diagnosis to death of patients with SCLC with BM, while CSS was from diagnosis to death due to cancer.

### 2.5 Statistical analysis

Descriptive statistics were generated and are expressed as frequency (percentage) for categorical variables. The Pearson’s chi-square test was used to compare categorical variables. Multivariable logistic regression models were used to evaluate the risk variables for BM in SCLC. Time-to-event data (OS, CSS) are expressed as Kaplan-Meier estimates, and log-rank tests were used to compare the differences in survival curves. Clinically important baseline variables or those that exhibited univariate relationships with the outcome were included in multivariate in order to assess the relationship between OS (CSS) outcomes and prognostic factors, a Cox proportional hazards regression model was used. Based on the number of available events, the included variables were prudently selected, to guarantee parsimony of final models. Using the marginal Cox model, lesion-level multivariate models were adjusted for patient effects, and insignificant variables were dropped through backward selection. The SEER*Stat software was used to retrieve data for the years 2010 to 2016. Kaplan-Meier and Log-rank analyses were carried out using GraphPad Prism 8.0 (GraphPad Software). SPSS software version 26.0 (15) was used for the statistical analyses, and a P-value of 0.05 was considered significant. Patients were randomly assigned to two cohorts (training and validation cohorts) with a 7:3 ratio. Nomograms were created using “rms” in R (version 3.6.1; http://www.r-project.org/) and evaluated using the Harrell’s C-index, receiver operating curve (ROC), calibration curve, and decision curve analysis based on the outcomes of the multivariate Cox proportional hazards regression model (DCA). For deep learning purposes, we performed Recursive partitioning analysis (RPA) to construct a decision tree model for risk stratification using the R package ‘rpart’. Patients were allocated into 3 groups: high-, intermediate- and low-risk groups for survival analysis and forest plot presentation.

## 3 Results

### 3.1 Patient demographics

We identified 4201 patients diagnosed with SCLC between 2010 and 2016. Baseline data for all patients were selected from the SEER program dataset. Patient demographics, tumor characteristics, and treatment information are provided in **Table 1**. Based on age distribution, two groups were assigned for the patients: <65 years (34.02%) and ≥65 years (65.92%). The proportion of women (52.44%) was slightly higher than that of men (47.55%). Based on patients’ racial characteristics, 3747 (89.19%) were white patients, 265(6.31%) were black patients, and 189 were other race patients (4.50%). The ICD-O-3 histology code was used to classify the tumor histology. Patients were grouped into five groups: SCLC, fusiform cell, oat cell carcinoma, intermediate cell, and combined small cell carcinoma, the latter constituting the largest proportion (91.95%). Moreover, single tumor site was more common (74.67% of patients) than multiple sites of the lung tumor. The grades were predominantly classified as I, II, III, and IV in 0.62%, 1.37%, 36.78%, and 61.82%, respectively, with the proportion being notably higher in grade IV. The compositions of patients with tumor sizes (in cm) 0≤x<3 3≤x<5, 5≤x<7, 7≤x<10, and ≥10 was 27.99%, 21.33%, 20.30%, 17.81%, and 7.81%, respectively. Based on recommendations for tumor nodule metastasis (TNM) staging from the American Joint Committee on Cancer (AJCC), T4 and N2 patients had the biggest proportion based on the T and N stage (31.54% and 49.68%, respectively). Most patients (85.43%, 77.31%) had accompanying brain and/or liver metastases. A significant number of patients were treated with surgery (90.15%) or chemotherapy (71.44%). As for radiation, there was a slight radiotherapy predominance (51.13%) versus no radiation (48.87%) in the radiation distribution.

### 3.2 Incidence of BM

After the exclusion of patients with unknown BM information, 780 (18.57%) were reported to exhibit BM. **Table 1** presents the results obtained from the univariate analysis between clinicopathological variables and incidence of BM. We found that sex (χ2 = 15.081, P<0.001), histologic type (χ2 = 12.772, P=0.012), tumor number (χ2 = 17.274, P<0.001), tumor size (χ2 = 45.297, P<0.001), T stage (χ2 = 60.658, P<0.001), liver metastasis (χ2 = 79.240, P<0.001), N stage (χ2 = 154.467, P<0.001), brain metastasis (χ2 = 15.043, P<0.001), radiation sequence with surgery (χ2 = 49.181, P<0.001), surgery (χ2 = 508.699, P<0.001), and radiation (χ2 = 18.496, P<0.001) all showed significant correlation with the incidence of BM. Thus, we included the abovementioned variables in the multivariate analysis, appropriately relaxed the criteria in combination with clinical significance, and included chemotherapy (χ2 = 3.668, P=0.055).

### 3.3 Risk factors for BM

The following clinicopathological characteristics were discovered to be BM risk factors that were statistically significant: female sex (OR=0.766, 95% CI=0.645–0.909, P=0.002), N1 (OR=0.507, 95% CI =0.268–0.957, P<0.001), and surgery (OR=0.766, 95% CI=0.645–0.909, P<0.001). These factors were significantly associated with lower BM risk, and patients with T2 and T3 had lower BM risk compared with patients with T0. In contrast, brain metastasis (OR=1.353, 95% CI=1.071–1.709, P=0.011), liver metastasis (OR=4.915, 95% CI=4.103–5.887, P<0.001), and chemotherapy (OR=1.362, 95% CI=1.108–1.674, P=0.003) were significantly correlated with higher BM risk, and patients with larger tumor size (5≤x<7 cm: OR=1.476, 95% CI=1.035–2.105, P=0.031; 7≤x<10 cm: OR=1.660, 95% CI=1.166–2.364, P=0.005) were highly associated with BM than those with smaller tumor size. Detailed data are shown in **Table 2**.

**Table 2:** Univariate and multivariable Logistic regression for developing BM among SCLC patients.

Characteristics	Univariate analysis		Multivariable analysis	
	χ2	P value	OR (95% CI)	P value
**Age group**	1.312	0.252		
<65			Reference	
≥65			0.967(0.806-1.161)	0.721
**Gender**	15.801	<0.001		
Male			Reference	
Female			0.766(0.645-0.909)	0.002
**Race**	2.108	0.349	NI	
Black				
White				
Other				
**Histologic type**	12.772	0.012		0.821
Small cell carcinoma, NOS			Reference	
Oat cell carcinoma			1.128(0.673-1.891)	0.646
Small cell carcinoma, fusiform cell			0.906(0.433-1.895)	0.794
Small cell carcinoma, intermediate cell			1.986(0.123-32.078)	0.629
Combined small cell carcinoma			2.012(0.381-10.621)	0.41
**Tumor number**	17.274	<0.001		
N=1			Reference	
N>1			0.872(0.705-1.079)	0.207
**Primary site**	6.833	0.233	NI	
Main bronchus				
Upper lobe, lung				
Middle lobe, lung				
Lower lobe, lung				
Overlapping leision of lung				
Lung, NOS				
**Laterality**	1.83	0.401	NI	
Bilateral				
Left-origin of primary				
Right-origin of primary				
**Grade**	4.849	0.183		0.478
I			Reference	
II			1.827(0.623-5.361)	0.272
III			0.424(0.091-1.971)	0.274
IV			1.020(0.851-1.223)	0.83
**Size group**	45.297	<0.001		0.002
0≤X<3			Reference	
3≤X<5			1.194(0.814-1.751)	0.365
5≤X<7			1.476(1.035-2.105)	0.031
7≤X<10			1.660(1.166-2.364)	0.005
X≥10			1.008(0.705-1.440)	0.965
**T**	60.658	<0.001		0.011
T1			Reference	
T2			0.568(0.324-0.996)	0.048
T3			0.577(0.349-0.955)	0.032
T4			0.821(0.497-1.357)	0.442
TX			0.796(0.487-1.301)	0.363
**N**	154.467	<0.001		<0.001
N0			Reference	
N1			0.507(0.268-0.957)	0.037
N2			0.853(0.443-1.643)	0.638
N3			1.038(0.576-1.871)	0.893
NX			1.794(0.978-3.288)	0.063
**Brain metastasis**	15.825	<0.001		
No			Reference	
Yes			1.353(1.071-1.709)	0.011
**Liver metastasis**	508.699	<0.001		
No			Reference	
Yes			4.915(4.103-5.887)	<0.001
**Radiation sequence with surgery**	15.043	0.001		0.241
No			Reference	
After			0.339(0.089-1.295)	0.114
Prior			0.309(0.079-1.215)	0.093
**Surgery**	79.24	<0.001		
No/Unknown			Reference	
Yes			0.278(0.142-0.543)	<0.001
**Radiation**	18.496	<0.001		
No/Unknown			Reference	
Yes			0.825(0.675-1.009)	0.061
**Chemotherapy**	3.668	0.055		
No/Unknown			Reference	
Yes			1.362(1.108-1.674)	0.003

### 3.4 Analysis of survival

**Figure 2:**
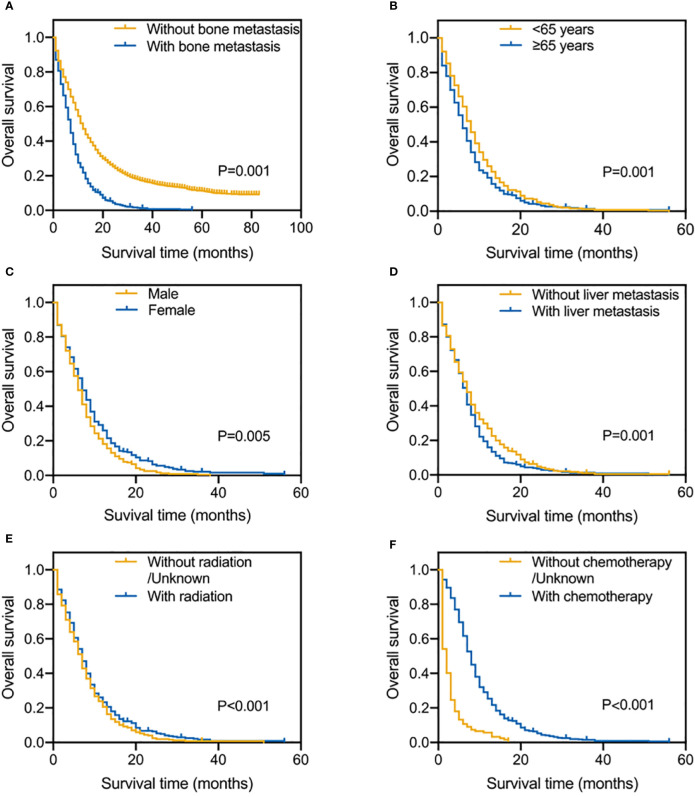
Kaplan-Meier survival curve analysis of SCLC patients with and without BM **(A)**; Risk factors for SCLC patients with BM by the Kaplan-Meier survival curve analysis: age **(B)**, gender **(C)**, liver metastasis **(D)**, radiation **(E)** and chemotherapy **(F)**.

**Figure 2A** shows the OS results for SCLC patients who had vs. who did not have BM. Patients with SCLC with BM had a shorter median survival time (MST) for OS (MST=6 months, 95% CI=5.441–6.559 months) than patients with SCLC without BM (MST=10 months, 95% CI=9.507–10.493 months). To calculate OS, Kaplan-Meier survival curves were utilized in different subgroups based on prognostic factors. Age, sex, liver metastasis, radiation, and chemotherapy were significantly associated with OS (**Figures 2B–F**). The results showed that SCLC patients with BM younger than 65 years old, female, without liver metastasis, receiving radiotherapy and chemotherapy have a better OS. Long rank tests and Kaplan-Meier survival curves were significant (P<0.05), while other variables did not exert significant effects on OS (P>0.05).

### 3.5 Prognostic factors for CSS and OS of BM patients

To determine prognostic indicators for OS and CSS, univariate and multivariate Cox proportional risk regression models were conducted, as shown in **Tables 3**, **4**. Age ≥65 (HR=1.248, 95% CI=1.042–1.495, P=0.015), female (HR=0.749, 95% CI=0.627–0.895, P=0.001), tumor size between 7≤X<10 (HR=1.606, 95% CI=1.219–2.117, P=0.001), N2-3 (HR=1.391, 95% CI=1.239–1.411, P=0.039; HR=1.485, 95% CI=1.291–1.573, P=0.007), liver metastasis (HR=1.392, 95% CI=1.166–1.652, P=0.001), radiotherapy (HR=0.805, 95% CI=0.661–0.979, P=0.03) as well as chemotherapy (HR=0.200, 95% CI=0.161–0.249, P=0.001) were independent prognostic factors affecting OS. Meanwhile, Age ≥65 (HR=1.320, 95% CI=1.081–1.613, P=0.006), female (HR=0.719, 95% CI=0.588–0.878, P=0.001), white race (HR=1.792, 95% CI=1.174–2.735, P=0.006), tumor number >1 (HR=0.049, 95% CI=0.021–0.111, P=0.001), tumor size between 7≤X<10 (HR=1.673, 95% CI=1.231–2.274, P=0.001), N3 (HR=1.570, 95% CI=1.075–2.293, P=0.019), liver metastasis (HR=1.351, 95% CI=1.107–1.649, P=0.003), radiotherapy (HR=0.769, 95% CI=0.614–0.964, P=0.022) and chemotherapy (HR=0.178, 95% CI=0.138–0.230, P=0.001) were independent prognostic factors for CSS.

**Table 3:** Univariate and multivariable Cox regression for overall survival among SCLC patients with BM.

Characteristics	Univatiate analysis		Mutivatiate analysis	
	HR (95% CI)	P value	HR (95% CI)	P value
Age group				
<65	Reference		Reference	
≥65	1.276(1.071-1.454)	0.003	1.248(1.042-1.495)	0.015
**Gender**				
Male	Reference		Reference	
Female	0.820(0.690-0.974)	0.0241	0.749(0.627-0.895)	0.001
**Race**				
Black	Reference		Reference	
White	1.100(0.764-1.582)	0.609	1.447(0.996-2.101)	0.052
Other	1.029(0.603-1.757)	0.916	1.253(0.716-2.193)	0.428
**Histologic type**			NI	
Small cell carcinoma, NOS	Reference			
Oat cell carcinoma	1.017(0.573-1.805)	0.952		
Small cell carcinoma, fusiform cell	14.801(2.047-107.007)	0.007		
Small cell carcinoma, intermediate cell	0.884(0.220-3.547)	0.862		
Combined small cell carcinoma	1.108(0.662-1.854)	0.694		
**Tumor number**			NI	
N=1	Reference			
N>1	0.933(0.748-1.166)	0.545		
**Primary site**			NI	
Main bronchus	Reference			
Upper lobe, lung	0.743(0.558-0.990)	0.042		
Middle lobe, lung	0.426(0.258-0.705)	0.001		
Lower lobe, lung	0.652(0.478-0.888)	0.006		
Overlapping leision of lung	0.407(0.163-1.018)	0.054		
Lung, NOS	0.735(0.486-1.112)	0.146		
**Laterality**			NI	
Bilateral	Reference			
Left-origin of primary	0.708(0.226-2.217)	0.554		
Right-origin of primary	0.722(0.231-2.258)	0.576		
**Grade**			NI	
I	Reference			
II	3.907(0.434-35.094)	0.224		
III	1.979(0.734-5.333)	0.177		
IV	1.995(0.743-5.352)	0.17		
Size group				
0≤X<3	Reference		Reference	
3≤X<5	0.980(0.766-1.255)	0.877	0.936(0.730-1.201)	0.607
5≤X<7	0.927(0.718-1.198)	0.564	1.122(0.866-1.454)	0.381
7≤X<10	1.350(1.032-1.768)	0.028	1.606(1.219-2.117)	0.001
X≥10	1.169(0.807-1.694)	0.406	1.145(0.783-1.674)	0.484
**T**			NI	
T1	Reference			
T2	0.978(0.709-1.349)	0.893		
T3	0.986(0.718-1.353)	0.931		
T4	1.221(0.904-1.650)	0.192		
TX	0.957(0.511-1.794)	0.893		
N				
N0	Reference		Reference	
N1	1.036(0.678-1.584)	0.868	1.187(0.308-0.911)	0.431
N2	1.391(1.023-1.891)	0.034	1.391(0.411-1.239)	0.039
N3	1.425(1.026-1.977)	0.034	1.573(0.485-1.291)	0.007
NX	1.796(0.880-3.665)	0.107	1.241(0.523-1.424)	0.558
Brain metastasis				
No	Reference		Reference	
Yes	0.964(0.777-1.196)	0.741	1.197(0.943-1.521)	0.138
Liver metastasis				
No	Reference		Reference	
Yes	1.336(1.126-1.586)	0.001	1.392(1.166-1.652)	0.001
**Radiation sequence with surgery**			NI	
No	Reference			
After	0.816(0.576-1.156)	0.253		
Prior	1.513(0.485-4.717)	0.475		
**Surgery**			NI	
No	Reference			
Yes	1.129(0.534-2.383)	0.751		
Radiation				
No	Reference		Reference	
Yes	0.695(0.583-0.829)	0.001	0.805(0.661-0.979)	0.03
Chemotherapy				
No	Reference		Reference	
Yes	0.225(0.183-0.276)	0.001	0.200(0.161-0.249)	0.001

**Table 4:** Univariate and multivariable Cox regression for cancer-specific survival among SCLC patients with BM.

Characteristics	Univatiate analysis		Mutivatiate analysis	
	HR (95% CI)	P value	HR (95% CI)	P value
Age group				
<65	Reference		Reference	
≥65	1.132(0.932-1.373)	0.211	1.320(1.081-1.613)	0.006
Gender				
Male	Reference		Reference	
Female	0.753(0.620-0.913)	0.004	0.719(0.588-0.878)	0.001
Race				
Black	Reference		Reference	
White	1.099(0.733-1.649)	0.646	1.792(1.174-2.735)	0.006
Other	0.964(0.525-1.772)	0.907	1.151(0.604-2.193)	0.667
**Histologic type**			NI	
Small cell carcinoma, NOS	Reference			
Oat cell carcinoma	1.188(0.651-2.164)	0.574		
Small cell carcinoma, fusiform cell	19.488(2.681-141.654)	0.003		
Small cell carcinoma, intermediate cell	1.150(0.286-4.618)	0.843		
Combined small cell carcinoma	1.207(0.694-2.099)	0.504		
Tumor number				
N=1	Reference		Reference	
N>1	0.057(0.025-0.127)	0.001	0.049(0.021-0.111)	0.001
**Primary site**			NI	
Main bronchus	Reference			
Upper lobe, lung	0.755(0.547-1.041)	0.087		
Middle lobe, lung	0.433(0.247-0.759)	0.003		
Lower lobe, lung	0.665(0.469-0.941)	0.021		
Overlapping leision of lung	0.519(0.205-1.309)	0.164		
Lung, NOS	0.730(0.458-1.166)	0.188		
**Laterality**			NI	
Bilateral	Reference			
Left-origin of primary	0.887(0.219-3.582)	0.867		
Right-origin of primary	0.860(0.213-3.466)	0.832		
**Grade**			NI	
I	Reference			
II	5.587(0.578-53.990)	0.137		
III	2.072(0.659-6.508)	0.212		
IV	2.215(0.709-6.919)	0.171		
Size group				
0≤X<3	Reference		Reference	
3≤X<5	0.993(0.754-1.307)	0.96	1.105(0.799-1.401)	0.69
5≤X<7	0.866(0.648-1.159)	0.335	1.179(0.877-1.586)	0.273
7≤X<10	1.411(1.047-1.902)	0.023	1.673(1.231-2.274)	0.001
X≥10	1.233(0.821-1.853)	0.311	1.162(0.766-1.764)	0.479
**T**			NI	
T1	Reference			
T2	1.120(0.773-1.623)	0.549		
T3	1.101(0.763-1.589)	0.605		
T4	1.345(0.947-1.910)	0.097		
TX	1.219(0.623-2.385)	0.563		
N				
N0	Reference		Reference	
N1	1.085(0.681-1.727)	0.731	1.050(0.650-1.694)	0.841
N2	1.385(0.984-1.948)	0.061	1.427(0.999-2.039)	0.05
N3	1.402(0.973-2.020)	0.069	1.570(1.075-2.293)	0.019
NX	1.460(0.617-3.452)	0.388	1.222(0.507-2.944)	0.654
Brain metastasis				
No	Reference		Reference	
Yes	1.095(0.867-1.381)	0.445	1.300(0.999-1.690)	0.05
Liver metastasis				
No	Reference		Reference	
Yes	1.465(1.208-1.776)	0.001	1.351(1.107-1.649)	0.003
**Radiation sequence with surgery**			NI	
No	Reference			
After	0.871(0.596-1.271)	0.474		
Prior	1.277(0.317-5.133)	0.731		
**Surgery**			NI	
No	Reference			
Yes	0.990(0.409-2.395)	0.983		
Radiation				
No	Reference		Reference	
Yes	0.694(0.570-0.846)	0.001	0.769(0.614-0.964)	0.022
Chemotherapy				
No	Reference		Reference	
Yes	0.235(0.186-0.297)	0.001	0.178(0.138-0.230)	0.001

### 3.6 Nomogram

**Figure 3:**
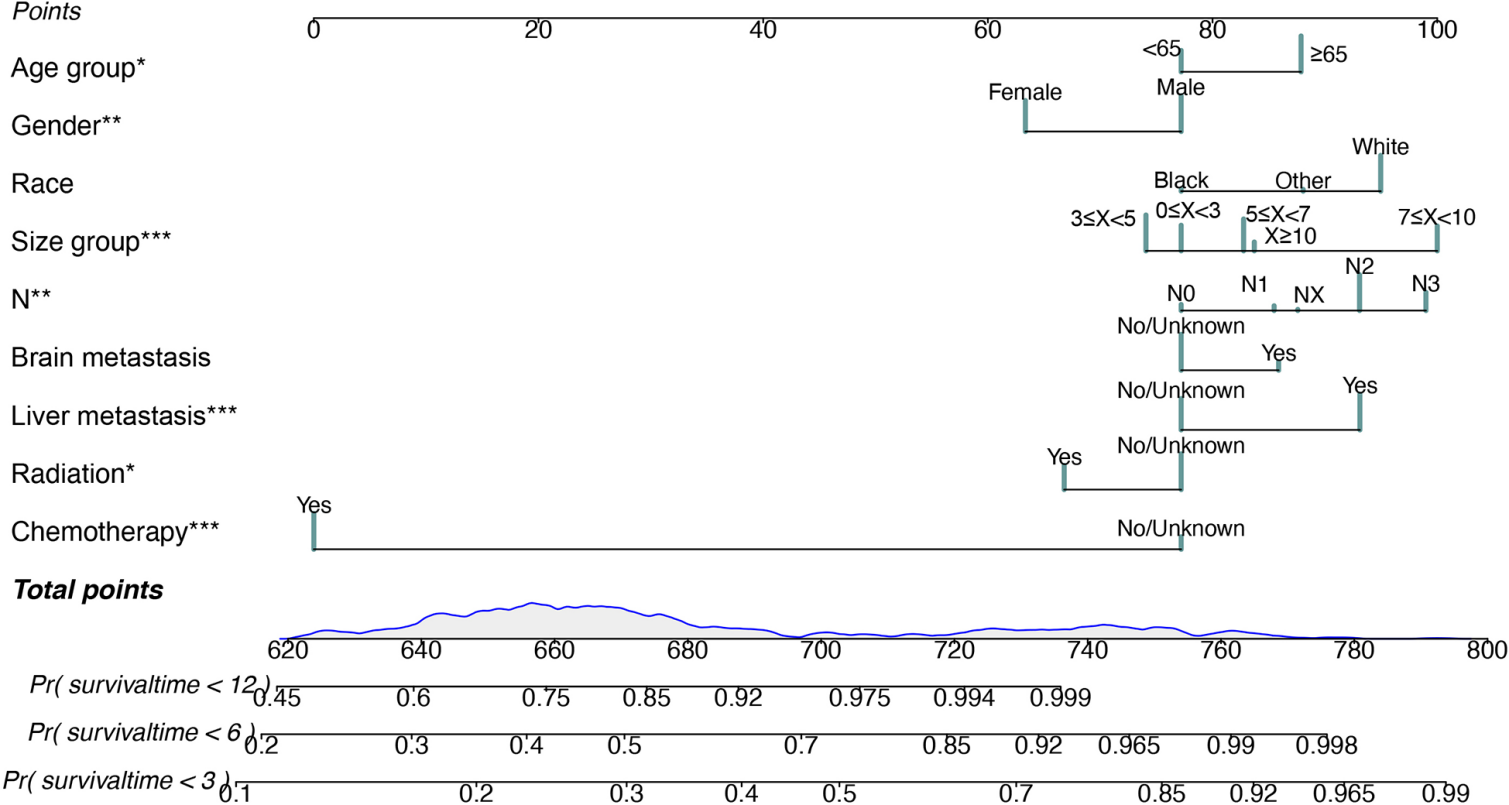
Nomogram for predicting 3-month, 6-month and 12-month overall survival in SCLC patients with BM. *P < 0.05, **P < 0.01, ***P < 0.001.

**Figure 4:**
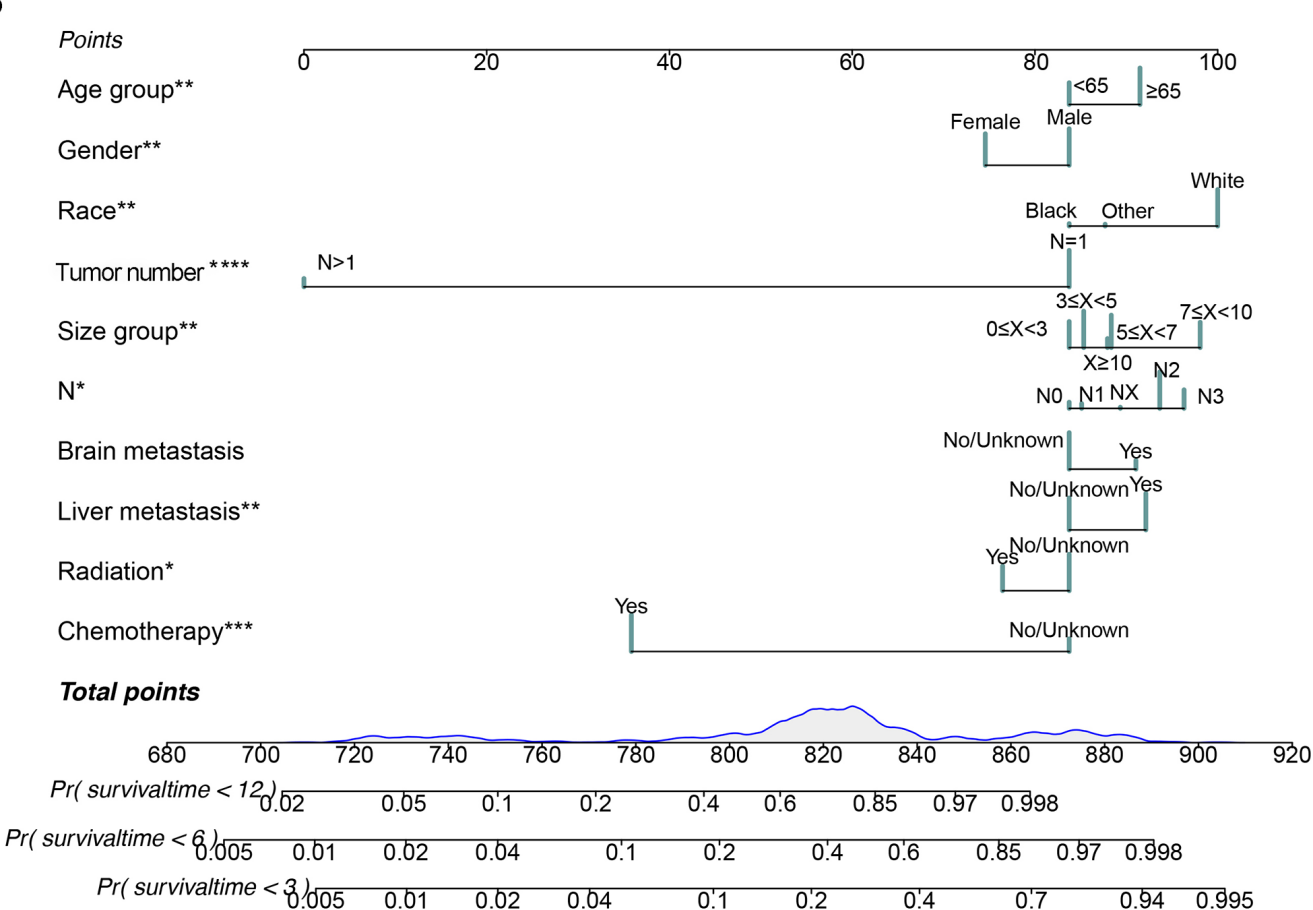
Nomogram for predicting 3-month, 6-month and 12-month cancer-specific survival in SCLC patients with BM. *P < 0.05, **P < 0.01, ***P < 0.001.

**Figure 5:**
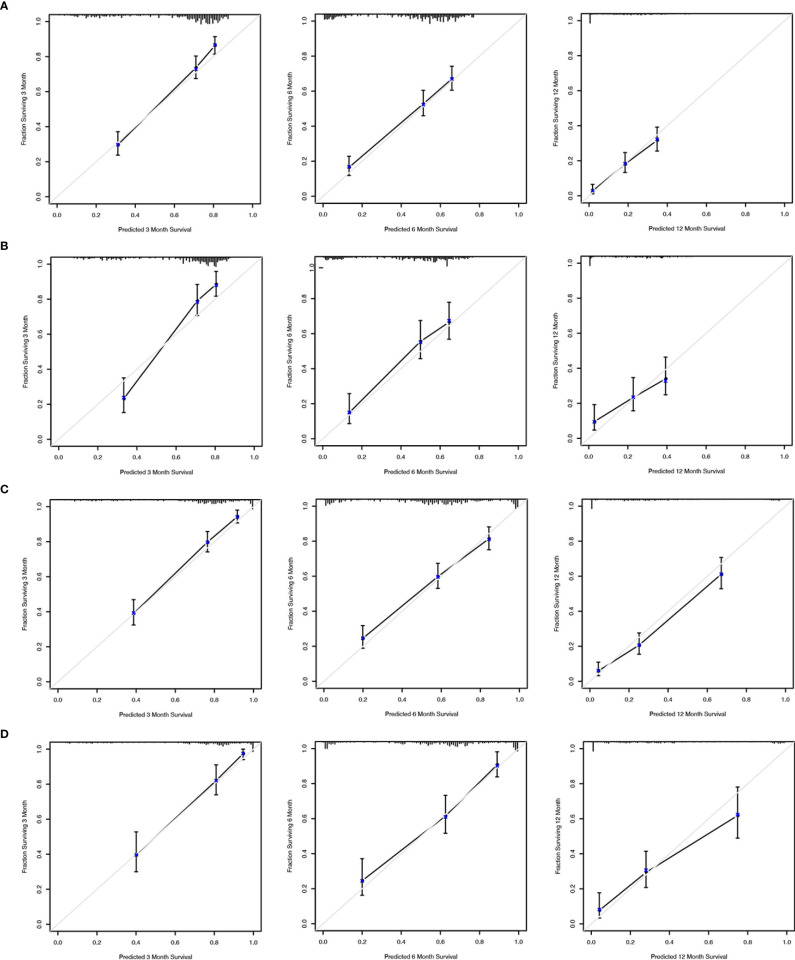
Calibration curves to predict 3-month, 6-month and 12-month overall survival of SCLC patients with BM in the training cohort **(A)** and validation cohort **(B)**. Calibration curves to predict cancer-specific survival in SCLC patients with BM in the training cohort **(C)** and validation cohort **(D)**.

**Figure 6:**
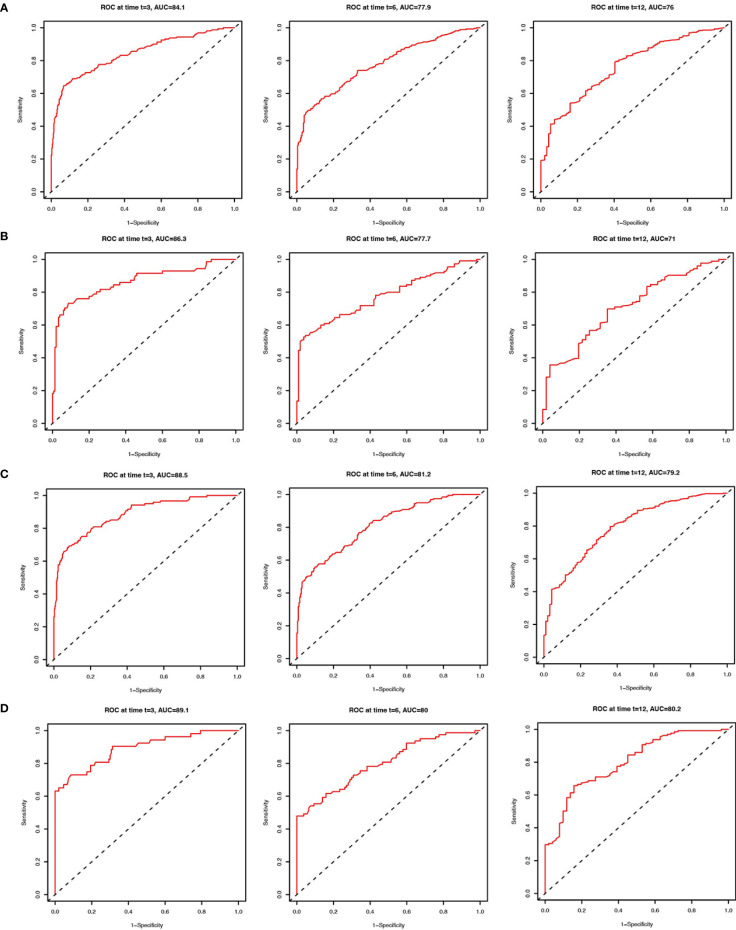
ROC curves to predict 3-month, 6-month and 12-month overall survival of SCLC patients with BM in the training cohort **(A)** and validation cohort **(B)**. ROC curves to predict cancer-specific survival in SCLC patients with BM in the training cohort **(C)** and validation cohort **(D)**.

**Figure 7:**
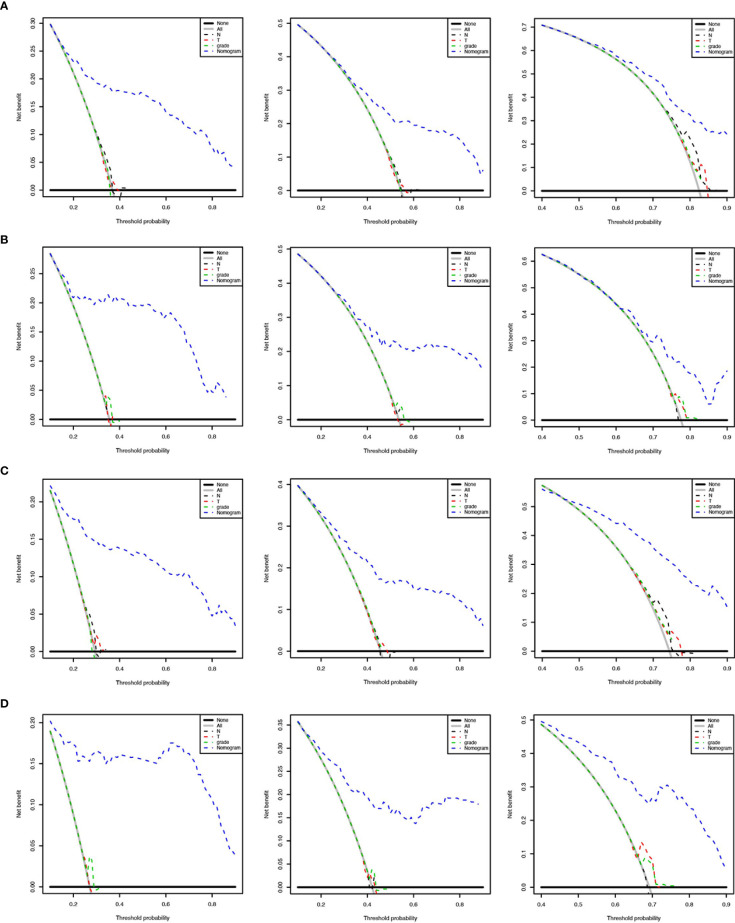
Decision curves to predict 3-month, 6-month and 12-month overall survival of SCLC patients with BM in the training cohort **(A)** and validation cohort **(B)**. Decision curves to predict cancer-specific survival in SCLC patients with BM in the training cohort **(C)** and validation cohort **(D)**.

**Figure 8:**
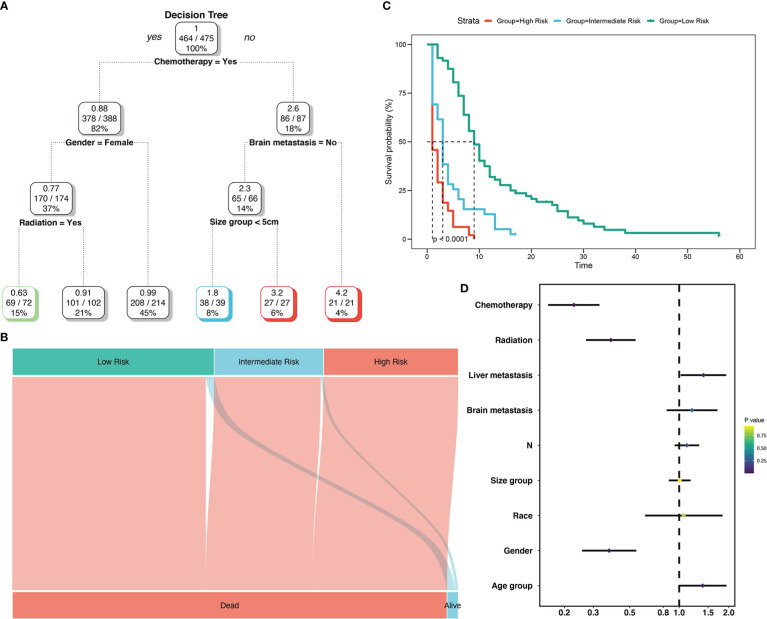
Decision tree analysis model for overall survival of SCLC patients with BM. Decision tree **(A)**; Decision tree analysis results into different risk groups **(B)**; Survival curves for different risk groups **(C)**; Forest map **(D)**.

We applied the nomogram model to further predict OS and CSS (**Figures 3**, **4**) in SCLC patients with BM. Gender, race, tumor size, age, N stage, brain metastasis, liver metastasis, radiation as well as chemotherapy were included in the nomogram for OS and CSS. C-indices for validation of OS as well as CSS predictions were 0.715 (95% CI, 0.702-0.728) and 0.769 (95% CI, 0.70-0.783), respectively. The 3-month, 6-month and 12-month OS and CSS calibration curves (**Figure 5**) revealed that the actual and predicted survival probabilities were in strong accordance with validation as well as training cohorts. Meanwhile, area under the curve (AUC) and ROC curves were developed through time. Overall, the values of AUCs for OS and CSS (**Figure 6A–D**) at different time points were around 0.7 in the training as well as validation cohorts, indicating that the OS and CSS nomograms were accurate and valid at different time points. Clinical applications were evaluated with DCA, and the DCA curves at different time points had greater net benefit in training as well as validation cohorts for the OS (**Figure 8**) and the CSS (**Figure 7**).

### 3.7 Decision tree

**Figure 9:**
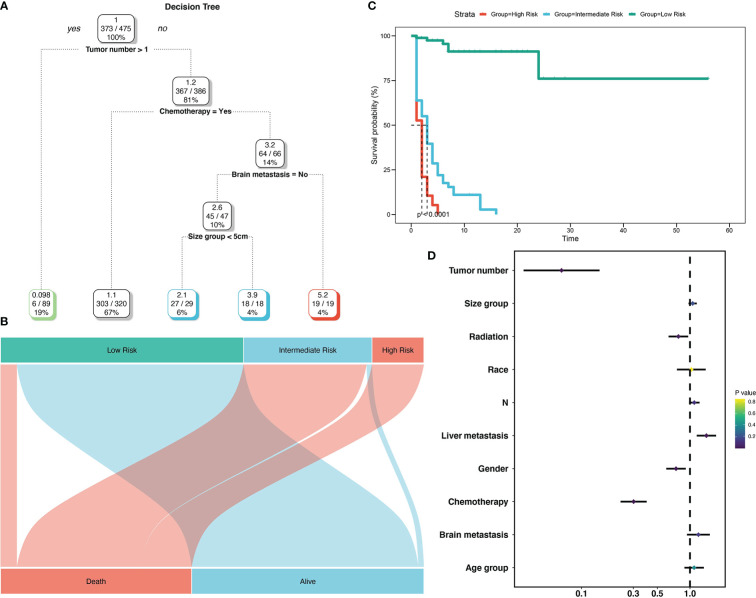
Decision tree analysis model for cancer-specific survival of SCLC patients with BM. Decision tree **(A)**; Decision tree analysis results into different risk groups **(B)**; Survival curves for different risk groups **(C)**; Forest map **(D)**.

To create decision rules influencing SCLC patients with BM, decision tree analysis incorporated every variable in the OS nomogram (**Figure 8**). First, chemotherapy was the most vital determinant because it was the 1^st^ level split of two initial branches of the classification tree. Gender and radiotherapy were the most vital determinants for 2^nd^ and 3^rd^ level splits, respectively. Furthermore, we classified SCLC patients with BM into three risk groups based on the decision tree results: high, intermediate, and low, with marked differences in survival analysis among the three risk groups. Forest plots represented the final Cox results. We used the same method for decision tree analysis of CSS (**Figure 9**). The results showed that the tumor number was the best determinant of CSS. Further analysis according to the guidelines revealed that a higher proportion of patients with tumor number >1 was older than 65 years (**Table 5**), leading to the possibility that patients with high tumor number were included in the low-risk group population. In addition, the survival time increased sequentially in high, intermediate as well as low risk groups. Cox results were represented as forest plots.

**Table 5:** Analysis of tumor number and associated variables in the CSS cohort.

	CSS Cohort		
	N=1	N>1	P value
**No. of Patients**	445	101	
**Age group (%)**			0.004
<65	353(79.32)	88(87.13)	
≥65	92(20.68)	13(12.87)	
**Gender (%)**			0.004
Male	193(43.37)	60(59.41)	
Female	252(56.63)	41(40.59)	
**Stage (%)**			0.093
I	3(0.67)	1(0.99)	
II	1(0.22)	0(0.00)	
III	150(33.71)	39(38.61)	
IV	291(65.39)	61(60.39)	
**Brain metastasis (%)**			0.072
No	353(79.33)	88(87.13)	
Yes	92(20.67)	13(12.87)	

## 4 Discussion

This cohort study is the largest (nearly 4201 patients) to use deep learning to predict BM in SCLC patients. Numerous studies have shown that BM is a high-risk factor for SCLC patients (14, 16). The occurrence of bone metastasis can cause compression of spinal cord, chronic bone pain, and pathological fractures among other disorders, thus worsening the life quality of patients (17). The SEER database was utilized in the current study to collect a large population of patients with SCLC to determine the incidence and some influencing factors for BM in patients with SCLC. Our research indicates that the combination of decision tree model and nomogram can be a convenient and successful method to evaluate BM prognosis in SCLC patients.

We found that BM occurred in 18.57% of 4201 patients diagnosed with SCLC. Previously, a prospective trial in Japan showed that BM prevalence in SCLC was found to be 40.4% (18). However, in Denmark, BM incidences was markedly low (16.7%), which was consistent with our results (19). In our findings, some risk factors were significantly correlated with BM development in patients with SCLC. Demographic variables (sex) and clinicopathological features include tumor size, T and N stages, brain and liver metastases, surgery, as well as chemotherapy. An earlier large-cohort study that looked at colorectal cancer patients as a whole population revealed a comparable pattern in terms of the relationship between various risk factors and patient BM development (20). These risk factors can effectively guide physicians to focus on which clinical factors have a higher risk of BM. BM is a poor prognostic factor in lung cancers. BM is a predictor of poor survival in NSCLC and SCLC. In SCLC, BM patients have also been shown to exhibit poor survival outcomes (19, 21, 22). In our study, we observed an MST of 6 months in SCLC patients with BM, relative to 10 months in those without BM. These results that BM has a marked and clinically significant poor effects on the prognosis of SCLC patients are consistent with those of previous studies.

Older age, male, white race, and liver metastasis were determined from the patient’s features to be independent prognostic factors affecting the prognosis of BM SCLC patients. Our study showed that the MST did make the difference between the two age groups in SCLC with BM, which was 5 months in older patient versus 7 months in younger patients. At the same time, SCLC is highly aggressive and progresses so rapidly that the OS time is reduced. Therefore, there is a need for physicians to focus on lung tumor patients exhibiting these basic prognostic factors.

The liver is a common site of distant metastasis in SCLC (21). In our study, there is a particularly surprising finding that patients with BM with liver metastasis have worse prognosis (MST was 6 months versus 7 months). This observed difference may support the close association between liver metastases and BM in SCLC. In an *in vitro* experiment, researchers reported that using methods adequately suppressed the liver metastatic potential of SCLC cells but concurrently promoted metastatic bone lesion progression, implying heterogeneity in metastatic effects (23). Therefore, further studies to assess probable mechanisms and explore relationships are required.

For BM SCLC patients, tumor size, tumor number, and N stage were found to be independent prognostic factors. Lymph node metastasis is a common form of metastasis in SCLC, and the high incidence is also associated with multiple metastasis and poor differentiation (24). By indirectly affecting prognosis of patient as described above, it indicates that N stage is related to patient prognosis. Surprisingly, there was a correlation between tumor size and prognosis of SCLC patients with BM, but a larger tumor was not correlated with the reference tumor size of <3 cm in their prognosis. These findings may be somewhat limited by the T stage; therefore, further evaluation focusing on these items is needed.

From a therapeutic point of view, the traditional treatment for SCLC patients with BM is mainly chemotherapy (25). We found that chemotherapy is a significant prognostic factor in SCLC patients with BM, which corroborated the findings of previous studies. The main therapeutic options are platinum-based, two-drug therapy, such as carboplatin or cisplatin plus etoposide, with the aim of managing symptoms and enhancing survival outcomes. In addition, a recent study found that adjuvant radiation therapy may be beneficial for SCLC patients with BM (26), implying that for SCLC patients with BM, radiation therapy may also be a possibility for treatment. Same as our results, the prognosis of SCLC patients with BM can be improved using radiotherapy.

If the above risk factors are found to exist in SCLC patients, we should be alert to the risk of BM and apply the model in this research to determine the necessity of further therapeutic interventions, and clinicians can predict the prognosis of patients in a timely and accurate manner. With the rational application of the guiding model, the nomogram can be further applied to assess the prognosis after the occurrence of BM, and the decision tree model can make further decisions based on clinical characteristics. Thus, in combination with the assessment results, more appropriate treatment and more optimal care can be provided for high-risk patients. Conversely, for low-risk patients, some treatments and tests can be appropriately adjusted or reduced, thus reducing the burden on the patient.

There are still some limitations in our study, so the disadvantages of the present meta-analysis should be discussed. First, a certain number of patients with incomplete and invalid information were excluded. Second, the SEER database only contains data from only four sites of diagnosis, and combined adrenal gland metastasis or other metastatic sites can occur in patients with SCLC (27). In terms of BM, there is no specific site information on BM in patients with SCLC with BM. Third, the SEER database lacks several crucial clinicopathological factors, such as information regarding adjuvant chemotherapy and surgery on metastasis, as well as driven mutations in SCLC, which deserve further study. Finally, the SEER database lacks specific diagnostic guidelines for BM, such as the examination of radionuclide imaging, especially for ECT and PET/CT.

## 5 Conclusions

Our findings form a basis for the applications of advanced deep learning techniques to accurately predict the development of BM in SCLC patients. If validated in prospective studies, the above models may be effective ways to predict the prognosis of SCLC with BM patients and could guide individualized diagnosis and treatment.

## Data availability statement

The raw data supporting the conclusions of this article will be made available by the authors, without undue reservation.

## Ethics statement

Ethics approval and consent not needed since the data was obtained from SEER database. No patients were recruited by us for this study.

## Author contributions

QC, HL, LZ and HLu carried out the data analysis and drafted the manuscript. FC collected data and performed the statistical analysis. YG, ZH, BW and AH participated in its design. JD, LJ and WH designed the study and reviewed the article. All authors have been actively involved in the drafting and critical revision of the manuscript, and each provided final approval of the version to be published.

## References

[1] Ashour Badawy A Khedr G Omar A Bae S Arafat W Grant S. Site of metastases as prognostic factors in unselected population of stage IV non-small cell lung cancer. Asian Pacific J Cancer Prev (2018) 19(7):1907–10. doi: 10.22034/APJCP.2018.19.7.1907 PMC6165640 30051671

[2] Chen W Zheng R Baade PD Zhang S Zeng H Bray F. Cancer statistics in China, 2015. CA Cancer J Clin (2016) 66(2):115–32. doi: 10.3322/caac.21338 26808342

[3] Govindan R Page N Morgensztern D Read W Tierney R Vlahiotis A. Changing epidemiology of small-cell lung cancer in the united states over the last 30 years: Analysis of the surveillance, epidemiologic, and end results database. J Clin Oncol (2006) 24(28):4539–44. doi: 10.1200/JCO.2005.04.4859 17008692

[4] Wang S Tang J Sun T Zheng X Li J Sun H. Survival changes in patients with small cell lung cancer and disparities between different sexes, socioeconomic statuses and ages. Sci Rep (2017) 7(1):1339. doi: 10.1038/s41598-017-01571-0 28465554 PMC5431017

[5] Anwar A Jafri F Ashraf S Jafri MAS Fanucchi M. Paraneoplastic syndromes in lung cancer and their management. Ann Transl Med (2019) 7(15):359. doi: 10.21037/atm.2019.04.86 31516905 PMC6712246

[6] Hernandez RK Wade SW Reich A Pirolli M Liede A Lyman GH. Incidence of bone metastases in patients with solid tumors: Analysis of oncology electronic medical records in the united states. BMC cancer (2018) 18(1):44–. doi: 10.1186/s12885-017-3922-0 PMC5756362 29306325

[7] Cai H Wang H Li Z Lin J Yu J. The prognostic analysis of different metastatic patterns in extensive-stage small-cell lung cancer patients: a large population-based study. Future Oncol (2018) 14(14):1397–407. doi: 10.2217/fon-2017-0706 29359568

[8] Bhattacharya IS Hoskin PJ. Stereotactic body radiotherapy for spinal and bone metastases. Clin Oncol (2015) 27(5):298–306. doi: 10.1016/j.clon.2015.01.030 25687175

[9] Byers LA Rudin CM. Small cell lung cancer: Where do we go from here? Cancer (2015) 121(5):664–72. doi: 10.1002/cncr.29098 PMC5497465 25336398

[10] Jett JR Schild SE Kesler KA Kalemkerian GP. Treatment of small cell lung cancer: Diagnosis and management of lung cancer, 3rd ed: American college of chest physicians evidence-based clinical practice guidelines. Chest (2013) 143(5 Suppl):e400S–e19S. doi: 10.1378/chest.12-2363 23649448

[11] Zhao H Ren D Liu H Chen J. Comparison and discussion of the treatment guidelines for small cell lung cancer. Thorac Cancer (2018) 9(7):769–74. doi: 10.1111/1759-7714.12765 PMC6026606 29770597

[12] Wood DE. National comprehensive cancer network (NCCN) clinical practice guidelines for lung cancer screening. Thorac Surg Clinics (2015) 25(2):185–97. doi: 10.1016/j.thorsurg.2014.12.003 25901562

[13] Cho YJ Cho YM Kim SH Shin KH Jung ST Kim HS. Clinical analysis of patients with skeletal metastasis of lung cancer. BMC Cancer (2019) 19(1):303. doi: 10.1186/s12885-019-5534-3 30943924 PMC6446278

[14] Gong L Xu L Yuan Z Wang Z Zhao L Wang P. Clinical outcome for small cell lung cancer patients with bone metastases at the time of diagnosis. J Bone Oncol (2019) 19:100265. doi: 10.1016/j.jbo.2019.100265 31763163 PMC6859228

[15] Stone GW Maehara A Lansky AJ de Bruyne B Cristea E Mintz GS. A prospective natural-history study of coronary atherosclerosis. New Engl J Med (2011) 364(3):226–35. doi: 10.1056/NEJMoa1002358 21247313

[16] Zhou Y Yu Q-F Peng A-F Tong W-L Liu J-M Liu Z-L. The risk factors of bone metastases in patients with lung cancer. Sci Rep (2017) 7(1):8970. doi: 10.1038/s41598-017-09650-y 28827719 PMC5567132

[17] Liu W-C Li Z-Q Luo Z-W Liao W-J Liu Z-L Liu J-M. Machine learning for the prediction of bone metastasis in patients with newly diagnosed thyroid cancer. Cancer Med (2021) 10(8):2802–11. doi: 10.1002/cam4.3776 PMC8026946 33709570

[18] Uei H Tokuhashi Y. Prognostic factors in patients with metastatic spine tumors derived from lung cancer–a novel scoring system for predicting life expectancy. World J Surg Oncol (2018) 16(1):131. doi: 10.1186/s12957-018-1439-x 29976208 PMC6034326

[19] Cetin K Christiansen CF Jacobsen JB Norgaard M Sorensen HT Riihimaki M. Bone metastasis, skeletal-related events, and mortality in lung cancer patients: A Danish population-based cohort study. Lung Cancer (2014) 86(2):247–54. doi: 10.1016/j.lungcan.2014.08.022 25240518

[20] Li X Hu W Sun H Gou H. Survival outcome and prognostic factors for colorectal cancer with synchronous bone metastasis: A population-based study. Clin Exp Metastasis (2021) 38(1):89–95. doi: 10.1007/s10585-020-10069-5 33420873

[21] Riihimaki M Hemminki A Fallah M Thomsen H Sundquist K Sundquist J. Metastatic sites and survival in lung cancer. Lung Cancer (2014) 86(1):78–84. doi: 10.1016/j.lungcan.2014.07.020 25130083

[22] Nakazawa K Kurishima K Tamura T Kagohashi K Ishikawa H Satoh H. Specific organ metastases and survival in small cell lung cancer. Oncol Lett (2012) 4(4):617–20. doi: 10.3892/ol.2012.792 PMC3506697 23205072

[23] Takeuchi S Fukuda K Arai S Nanjo S Kita K Yamada T. Organ-specific efficacy of HSP90 inhibitor in multiple-organ metastasis model of chemorefractory small cell lung cancer. Int J Cancer (2016) 138(5):1281–9. doi: 10.1002/ijc.29858 26379118

[24] Lohinai Z Megyesfalvi Z Suda K Harko T Ren S Moldvay J. Comparative expression analysis in small cell lung carcinoma reveals neuroendocrine pattern change in primary tumor versus lymph node metastases. Transl Lung Cancer Res (2019) 8(6):938–50. doi: 10.21037/tlcr.2019.11.30 PMC6976364 32010572

[25] Wu S Pan Y Mao Y Chen Y He Y. Current progress and mechanisms of bone metastasis in lung cancer: A narrative review. Transl Lung Cancer Res (2021) 10(1):439–51. doi: 10.21037/tlcr-20-835 PMC7867745 33569325

[26] Gensheimer MF Loo BW Jr. Optimal radiation therapy for small cell lung cancer. Curr Treat Options Oncol (2017) 18(4):21. doi: 10.1007/s11864-017-0467-z 28391424

[27] Li J Liu F Yu H Zhao C Li Z Wang H. Different distant metastasis patterns based on tumor size could be found in extensive-stage small cell lung cancer patients: A large, population-based SEER study. PeerJ (2019) 7:e8163. doi: 10.7717/peerj.8163 31824772 PMC6896937

